# Supplementary material for: Content-rich biological network constructed by mining PubMed abstracts
Source: BMC Bioinformatics. 2004 Oct 8;5:147. doi: 10.1186/1471-2105-5-147 (PMC528731; doi:10.1186/1471-2105-5-147)
Supplement: Additional File 2 — The original results of the above study (non-essential files are deleted to keep the file size under the limit set by BMC bioinformatics). [file 1471-2105-5-147-S2.bz2 › chilibotAdditionalFile2/dip20/336ID10768840E3972/html/TNFSF8_TNFRSF8.html]

 


 **TNFSF8** and **TNFRSF8** 
  
Found 158 abstracts in PubMed, retrieved 20.  
 

 What does Google say? 
 PDF only 
| .edu only 

---

**Interactive relationship** (e.g. stimulation, inhibition, etc)

**Neutral relationship**- they activate HRS in vitro through CD30L  [ **TNFSF8** ]  CD30  [ **TNFRSF8** ]  interaction.  Ref: 12358914 Br J Haematol, 2002
- Characterization of the immunological properties of vCD30 has uncovered a role of CD30  [ **TNFRSF8** ]  CD30L  [ **TNFSF8** ]  interactions in the generation of inflammatory responses.  Ref: 12235215 J Exp Med, 2002
- The pleiotropic functions of CD30  [ **TNFRSF8** ]  are initiated by interaction of CD30  [ **TNFRSF8** ]  expressing cells with other immune competent cells expressing CD30  [ **TNFRSF8** ]  L  [ **TNFSF8** ]  and providing the signals for modulation of effector cell activity.  Ref: 12473266 Cell Immunol, 2002
- Comparison of effects of ligation with CD30L  [ **TNFSF8** ]  and anti CD30  [ **TNFRSF8** ]  agonistic antibody HeFi 1 revealed higher efficacy for CD30L  [ **TNFSF8** ]  in these ALCL lines.  Ref: 12855655 Clin Cancer Res, 2003
- However, CD30  [ **TNFRSF8** ]  is cleaved at the cell surface by tumor necrosis factor  [ **TNFSF8** ]  alpha converting enzyme TACE.  Ref: 11857410 Int J Cancer, 2002
- Direct sequencing analysis of the CD30  [ **TNFRSF8** ]  and CD30L  [ **TNFSF8** ]  genes revealed four polymorphisms one in the CD30  [ **TNFRSF8** ]  gene 201G A from the transcription start site, and three in the CD30L  [ **TNFSF8** ]  gene CA repeat in the promoter, 276G A in the exon 3T C in the intron 3 IVS3 73T C.  Ref: 11960307 Genes Immun, 2002
- Furthermore, a number of Th2 associated markers, including CD30  [ **TNFRSF8** ] , IL 4 and GATA 3, were expressed by residual T cells derived from CD30L  [ **TNFSF8** ]  AML but not from CD30L  [ **TNFSF8** ]  AML, in which the presence of the Th1 associated marker LAG 3 was documented in some cases.  Ref: 11918534 Br J Haematol, 2002
- Co expression of CD30  [ **TNFRSF8** ]  ligand  [ **TNFSF8** ]  and interleukin 4 IL 4 receptors by acute myeloid leukaemia blasts is associated with the expansion of IL 4 producing CD30  [ **TNFRSF8** ]  normal T cells.  Ref: 11918534 Br J Haematol, 2002
- We report that in H RS cells overexpression of CD30  [ **TNFRSF8** ]  leads to self aggregation, recruitment of TRAF2 and TRAF5, and NF kappaB activation, independent of CD30  [ **TNFRSF8** ]  ligand  [ **TNFSF8** ] .  Ref: 11971184 Oncogene, 2002
- The activation antigen CD30  [ **TNFRSF8** ]  Ki 1  [ **TNFRSF8** ]  which is expressed by the lymphocytes T and B is related to the nerve growth factor NFGR like the receptor of the tumor necrosis factor  [ **TNFSF8** ]  TNF and is expressed in the group of large cell anaplastic lymphoma ACLC and Hodgkin s lymphoma and in non Hodgkin s lymphoma and within the T cells of infectious Mononucleosis.  Ref: 12730648 Infez Med< MedlineTA>Infez Med, 2002
- Proliferation of CD30  [ **TNFRSF8** ]  T Helper 2 Lymphoma Cells Can Be Inhibited by CD30  [ **TNFRSF8** ]  Receptor Cross Linking with Recombinant CD30  [ **TNFRSF8** ]  Ligand  [ **TNFSF8** ] .  Ref: 12855655 Clin Cancer Res, 2003
- PURPOSE Cutaneous anaplastic large cell lymphomas ALCLs are characterized by the expression of CD30  [ **TNFRSF8** ] , spontaneous regression of skin lesions, and increased concentration of CD30  [ **TNFRSF8** ]  ligand  [ **TNFSF8** ]  CD30L  [ **TNFSF8** ] .  Ref: 12855655 Clin Cancer Res, 2003

**Non-interactive relationship** (e.g. studied together, co-existance, homology, etc.)

- In addition the absence of CD30  [ **TNFRSF8** ]  L  [ **TNFSF8** ]  abolished clonal contraction after primary expansion and interfered with secondary expansion upon boosting.  Ref: 12538158 Ann N Y Acad Sci, 2002
- Association study between CD30  [ **TNFRSF8** ]  and CD30  [ **TNFRSF8** ]  ligand  [ **TNFSF8** ]  genes and type 1 diabetes in the Japanese population.  Ref: 11960307 Genes Immun, 2002
- CD30  [ **TNFRSF8** ] , a member of the tumor necrosis factor  [ **TNFSF8** ]  receptor TNFR family, is a characteristic cell surface receptor for activated T cells and the malignant cells of Hodgkin s disease HD, anaplastic large cell lymphoma ALCL and a few other non Hodgkin s lymphomas.  Ref: 12389614 Leuk Lymphoma, 2002
- Co expression of interleukin 6 IL 6 and interleukin 6 receptor IL 6R in thyroid nodules is associated with co expression of CD30  [ **TNFRSF8** ]  ligand  [ **TNFSF8** ]  CD30  [ **TNFRSF8** ]  receptor.  Ref: 12553555 J Endocrinol Invest, 2002
- Based on our recent data of CD30  [ **TNFRSF8** ]  ligand  [ **TNFSF8** ]  CD30L  [ **TNFSF8** ]  CD30  [ **TNFRSF8** ]  receptor CD30  [ **TNFRSF8** ]  in these nodules and on the knowledge that this signal stimulates IL 6 production in non thyroid neoplasms, we wanted to evaluate the immunocytochemical expression of these 2 ligand receptor systems in a large archival series of paraffin embedded specimens.  Ref: 12553555 J Endocrinol Invest, 2002
- Pleiotropic signal transduction mediated by human CD30  [ **TNFRSF8** ]  a member of the tumor necrosis factor  [ **TNFSF8** ]  receptor TNFR family.  Ref: 12389614 Leuk Lymphoma, 2002
- We conclude that only in a subset of both benign and malignant thyroid nodules the IL 6 IL 6R signal could be induced by the CD30L  [ **TNFSF8** ]  CD30  [ **TNFRSF8** ] .  Ref: 12553555 J Endocrinol Invest, 2002
- Cowpox virus encodes a fifth member of the tumor necrosis factor  [ **TNFSF8** ]  receptor family a soluble, secreted CD30  [ **TNFRSF8** ]  homologue.  Ref: 12034885 Proc Natl Acad Sci U S A, 2002
- The purpose of this study was to determine whether CD30  [ **TNFRSF8** ]  or CD30L  [ **TNFSF8** ]  genes serve as a novel susceptibility gene for type 1 diabetes in humans.  Ref: 11960307 Genes Immun, 2002
- Co expression of IL 6 and IL 6R in the epithelial follicular cells was observed solely in CD30L  [ **TNFSF8** ]  CD30  [ **TNFRSF8** ]  positive nodules 5 15 33% oncocytic adenomas.  Ref: 12553555 J Endocrinol Invest, 2002
- All 3 non metastasized MTC were IL 6 IL 6R negative, and 1 3 was CD30L  [ **TNFSF8** ]  positive CD30  [ **TNFRSF8** ]  negative.  Ref: 12553555 J Endocrinol Invest, 2002
- Plasma levels of cytomegalovirus CMV specific immunoglobulin G IgG, soluble s CD30  [ **TNFRSF8** ] , sCD26 dipeptidyl peptidase IV DPP IV enzyme activity, and tumor necrosis factor  [ **TNFSF8** ]  receptor I TNFR I were assessed in human immunodeficiency virus HIV infected patients who experienced CMV retinitis CMVR as an immune restoration disease IRD during their first 6 months of highly active antiretroviral therapy HAART and in CMV seropositive, HIV infected patients with similar baseline CD4 T cell counts who had uneventful immune reconstitution.  Ref: 12085331 J Infect Dis, 2002
- In the normal thyroid tissue, colloid nodules, and another subset of benign and malignant thyroid nodules, IL 6 expression is under control of signals other than CD30L  [ **TNFSF8** ]  CD30  [ **TNFRSF8** ] .  Ref: 12553555 J Endocrinol Invest, 2002
- The notion that IL 4 was able to enhance in vitro proliferation of CD30L  [ **TNFSF8** ]  IL 4R purified leukaemic blasts suggests that the selective interaction of IL 4 producing CD30  [ **TNFRSF8** ]  T cells with CD30L  [ **TNFSF8** ]  leukaemic progenitors may have a role in the progression of this particular AML subset.  Ref: 11918534 Br J Haematol, 2002
- Unlike dendritic cells, the CD4 CD3 cells have little CD80 or CD86 expression but do express high levels of the TNF ligands, OX40 ligand and CD30  [ **TNFRSF8** ]  ligand  [ **TNFSF8** ] .  Ref: 12753741 Immunity, 2003
- 14 15 FTC and 5 6 ATC were CD30L  [ **TNFSF8** ]  CD30  [ **TNFRSF8** ]  negative.  Ref: 12553555 J Endocrinol Invest, 2002
- Characterization of the murine CD30  [ **TNFRSF8** ]  ligand  [ **TNFSF8** ]  CD153  [ **TNFSF8** ]  gene gene structure and expression.  Ref: 12392508 Tissue Antigens, 2002
- CD30  [ **TNFRSF8** ]  ligand  [ **TNFSF8** ]  CD30L  [ **TNFSF8** ] , but not its cognate receptor CD30  [ **TNFRSF8** ] , is frequently expressed on acute myeloid leukaemia AML blasts.  Ref: 11918534 Br J Haematol, 2002
- We hypothesize that CD30  [ **TNFRSF8** ]  CD30L  [ **TNFSF8** ]  interactions explain the unusual clinical behavior of cutaneous ALCLs.  Ref: 12855655 Clin Cancer Res, 2003
- The production of IL 4 in the absence of interferon gamma IFN gamma was also detected in CD3 CD30  [ **TNFRSF8** ]  T cells from CD30L  [ **TNFSF8** ]  AML.  Ref: 11918534 Br J Haematol, 2002
- Creating CD30  [ **TNFRSF8** ]  L  [ **TNFSF8** ]  deficient mice and studying CD8 CTL activation and memory responses, it was observed that the absence of CD30  [ **TNFRSF8** ]  L  [ **TNFSF8** ]  resulted in diminished primary clonal expansion of CD8 cells.  Ref: 12538158 Ann N Y Acad Sci, 2002
- The immunoregulatory roles of interleukin 2 IL 2, IL 4, IL 10, gamma interferon IFN gamma, tumor necrosis factor  [ **TNFSF8** ]  alpha TNF alpha, the soluble form of the IL 2 receptor sIL 2R, and the soluble form of CD30  [ **TNFRSF8** ]  sCD30 were evaluated in patients with hepatitis B virus HBV infection.  Ref: 12414777 Clin Diagn Lab Immunol, 2002
- IL 6 staining associated to absent expression of CD30L  [ **TNFSF8** ]  and CD30  [ **TNFRSF8** ]  was observed in 7 follicular adenomas all belonging to variants different from toxic and hyalinizing trabecular, 2 oncocytic adenomas, 5 of the 30 colloid nodules and 2 normal thyroids.  Ref: 12553555 J Endocrinol Invest, 2002
- CD153  [ **TNFSF8** ]  CD30  [ **TNFRSF8** ]  ligand  [ **TNFSF8** ]  has been described as a 40 kDa type II transmembrane glycoprotein belonging to the TNF superfamily and is expressed primarily by activated T cells, B cells and monocytes.  Ref: 12392508 Tissue Antigens, 2002
- We show that vCD30 is a 12 kD secreted protein that not only binds CD30L  [ **TNFSF8** ]  with high affinity and prevents its interaction with CD30  [ **TNFRSF8** ] , but it also induces reverse signaling in cells expressing CD30L  [ **TNFSF8** ] . vCD30 blocked the generation of interferon gamma producing cells in vitro and was a potent inhibitor of T helper cell Th 1 but not Th2 mediated inflammation in vivo.  Ref: 12235215 J Exp Med, 2002
- In conclusion, we could not find significant association between CD30  [ **TNFRSF8** ]  or CD30L  [ **TNFSF8** ]  genes and type 1 diabetes, but CA 9 allele in the promotor or T allele of 73T C in intron 3 in CD30L  [ **TNFSF8** ]  gene might play a minor role in the pathogenesis of type 1 diabetes, only in the Japanese female population.  Ref: 11960307 Genes Immun, 2002
- In a cell kinetic analysis we found that ALCL cell growth is effectively inhibited by CD30L  [ **TNFSF8** ]  but only in those cell lines expressing CD30  [ **TNFRSF8** ]  molecules in sufficient amounts on the cell surface.  Ref: 12855655 Clin Cancer Res, 2003
- We screened CD30  [ **TNFRSF8** ]  and CD30L  [ **TNFSF8** ]  genes for polymorphisms in Japanese patients with type 1 diabetes and control subjects.  Ref: 11960307 Genes Immun, 2002
- A vCD30Fc fusion protein binds CD153  [ **TNFSF8** ]  CD30  [ **TNFRSF8** ]  ligand  [ **TNFSF8** ]  specifically, and it completely inhibits CD153  [ **TNFSF8** ]  CD30  [ **TNFRSF8** ]  interactions.  Ref: 12034885 Proc Natl Acad Sci U S A, 2002
- Association studies revealed no association between the CD30  [ **TNFRSF8** ]  and CD30L  [ **TNFSF8** ]  genes and type 1 diabetes in the whole population.  Ref: 11960307 Genes Immun, 2002
- CD30  [ **TNFRSF8** ]  CD30  [ **TNFRSF8** ]  ligand  [ **TNFSF8** ]  CD30L  [ **TNFSF8** ]  signal transduction appears to protect against autoimmune diabetes by preventing expansion of autoreactive T cells and suppressing Th1 cytokine response.  Ref: 11960307 Genes Immun, 2002
- Of the 6 tumors arising from the parafollicular C cells medullary thyroid cancer, MTC, all 3 that had metastasized were CD30L  [ **TNFSF8** ]  CD30  [ **TNFRSF8** ]  IL 6 positive and IL 6R negative.  Ref: 12553555 J Endocrinol Invest, 2002
- In another oncocytic adenoma, another 4 conventional PTC and another 7 non conventional PTC CD30L  [ **TNFSF8** ]  CD30  [ **TNFRSF8** ]  expression was associated to expression of IL 6 only.  Ref: 12553555 J Endocrinol Invest, 2002
